# Actionable genetic variants in 4,198 Scottish participants from the Orkney and Shetland founder populations and implementation of return of results

**DOI:** 10.1016/j.ajhg.2025.02.018

**Published:** 2025-03-14

**Authors:** Shona M. Kerr, Lucija Klaric, Marisa D. Muckian, Kiera Johnston, Camilla Drake, Mihail Halachev, Emma Cowan, Lesley Snadden, John Dean, Sean L. Zheng, Prisca K. Thami, James S. Ware, Gannie Tzoneva, Alan R. Shuldiner, Zosia Miedzybrodzka, James F. Wilson

**Affiliations:** 1 MRC Human Genetics Unit, University of Edinburgh, Institute of Genetics and Cancer, Western General Hospital, Crewe Road, Edinburgh EH4 2XU, UK; 2 Centre for Global Health Research, Usher Institute, University of Edinburgh, Teviot Place, Edinburgh EH8 9AG, UK; 3 Department of Infectious Disease Epidemiology, London School of Hygiene & Tropical Medicine, Keppel Street, London WC1E 7HT, UK; 4 Department of Medical Genetics, Ashgrove House, NHS Grampian, Aberdeen AB25 2ZA, UK; 5 Medical Genetics Group, University of Aberdeen, Polwarth Building, Aberdeen AB25 2ZD, UK; 6 National Heart and Lung Institute, Imperial College London, London, UK; 7 MRC Laboratory of Medical Sciences, Imperial College London, London, UK; 8 Royal Brompton & Harefield Hospitals, Guy’s and St. Thomas’ NHS Foundation Trust, London, UK; 9 Regeneron Genetics Center, Tarrytown, NY, USA; 10 Centre for Genomic and Experimental Medicine, University of Edinburgh, Institute of Genetics and Cancer, Western General Hospital, Crewe Road, Edinburgh EH4 2XU, UK

**Keywords:** return of results, actionable variant, founder effect, exomes, Orkney, Shetland

## Abstract

The benefits of returning clinically actionable genetic results to participants in research cohorts are accruing, yet such a genome-first approach is challenging. Here, we describe the implementation of return of such results in two founder populations from Scotland. Between 2005 and 2015, we recruited >4,000 adults with grandparents from Orkney and Shetland into the Viking Genes research cohort. The return of genetic data was not offered at baseline, but in 2023, we sent invitations to participants for consent to return of actionable genetic findings. We generated exome sequence data from 4,198 participants and used the American College of Medical Genetics and Genomics (ACMG) v.3.2 list of 81 genes, ClinVar review, and pathogenicity status, plus manual curation, to develop a pipeline to identify potentially actionable variants. We identified 104 individuals (2.5%) with 108 actionable genotypes at 39 variants in 23 genes and validated these. Working with the NHS Clinical Genetics service, which provided genetic counseling and clinical verification of the research results, and after expert clinical review, we notified 64 consenting participants (or their next of kin) of their actionable genotypes. Ten actionable variants across seven genes (*BRCA1*, *BRCA2*, *ATP7B*, *TTN*, *KCNH2*, *MUTYH*, and *GAA*) have risen 50- to >3,000-fold in frequency through genetic drift in ancestral island localities. Viking Genes is one of the first UK research cohorts to return actionable findings, providing an ethical and logistical exemplar of return of results. The genetic structure in the Northern Isles of Scotland with multiple founder effects provides a unique opportunity for a tailored approach to disease prevention through genetic screening.

## Teaser

We identified actionable variants in research cohort volunteers and implemented the return of results to participants. We detail this process, from exome sequencing through pipeline development, variant review, ethics, and outcomes toward disease prevention in identified individuals and their families. Multiple founder effects suggest an opportunity for population-wide genetic screening.

## Introduction

Where exome or genome sequencing is clinically indicated, the opportunity arises to report additional incidental findings with the aim of diagnosis and pre-emptive clinical intervention. The American College of Medical Genetics and Genomics (ACMG) has led this approach and annually updates a benchmark list of genes for reporting.^1^ The v.3.2 list has 81 genes, which meet strict criteria: there must be strong evidence that certain variants within the gene cause disease and there must be effective medical intervention (for example, screening or medication) that can be offered. The great majority of the genes are associated with inherited cancer predisposition, cardiovascular risk, or metabolic diseases. These conditions are mostly inherited in an autosomal dominant fashion, but variants in genes causing autosomal recessive disease, which are only actionable if homozygous or compound heterozygous, are also present in the ACMG v.3.2 list. Although there has not yet been a universal international adoption of this approach, the ACMG list is the most widely used.

The consent obtained from participants in many research cohorts to date, including the UK Biobank (UKB),^2^ does not allow the return of results (RoR) about actionable variants (AVs) or genotypes. However, some newer US cohorts, such as All of Us*,* are committed to making their research results, including hereditary disease risk, accessible to participants.^3^ Geisinger’s MyCode Community Health Initiative provides a model for “genome-first” care,^4^ and an international policy on the RoR acknowledges the potential medical benefits to the individuals who are participating in genomics research.^5^

The populations of the Northern Isles of Scotland—the Orkney and Shetland archipelagos—are the most genetically isolated of all British and Irish populations, with the highest degree of Norse admixture and an enrichment of rare and low-frequency functional variants.^6^^,^^7^^,^^8^^,^^9^ Viking Genes (viking.ed.ac.uk) comprises a set of Northern and Western Isles population cohort studies aiming to explore the genetic causes of disease—the Orkney Complex Disease Study (ORCADES) and VIKING I, II, and III. ORCADES^9^ and VIKING I^10^ together contain a rich data resource of more than 4,000 deeply phenotyped and exome-sequenced research subjects, nearly all with three or four grandparents from Orkney or Shetland, respectively. In total, Viking Genes has over 10,000 participants, all of whom consented to the analysis of their DNA and to be contacted at a later date in connection with future ethically approved studies. We have previously reported in the literature founder variants in *KCNH2*, *BRCA1*, and *BRCA2* in Shetland and Orkney populations,^10^^,^^11^^,^^12^ with variants tracing back to individual island ancestries.

In Viking Genes, all cohort members were offered the option of consent to return of actionable genetic results. Building on a method developed by Kelly et al.^13^ to screen 130,048 adults in the Geisinger MyCode project, we used the ACMG v.3.2 list^1^ and variant classification information from the public database ClinVar^14^ to code automated filters to identify potential AVs within 4,198 members of the Viking Genes cohort. Here, we describe the analysis pipeline and summarize actionable results, the process of returning these results, and some challenges associated with the implementation of these procedures at scale. We provide evidence of multiple drifted actionable founder variants that could form the basis for a future targeted Northern Isles population genetic screening program.

## Subjects and methods

### Research volunteer recruitment and DNA collection

Eligible participants were recruited to Viking Genes, NHS Lothian South East Scotland Research Ethics Committee ref. 19/SS/0104. Research participants gave informed consent for research procedures including DNA sequencing (mandatory) and return of actionable results (optional). Viking Genes comprises three cohort studies—the ORCADES, VIKING I, and VIKING II/III. Recruitment to ORCADES took place from 2005 to 2011^11^ and to Viking I (Viking Health Study – Shetland) from 2013 to 2015.^10^ The mean age of living participants as of December 31, 2024, is 63.3 years. Blood or occasionally saliva samples from participants were collected, processed, and stored using standard operating procedures and managed through a laboratory information management system at the Edinburgh Clinical Research Facility, University of Edinburgh. Recruitment to VIKING II and VIKING III took place online from January 2020 to March 2023.^15^

### Viking Genes pedigree information

The records of the births, marriages, and deaths in Orkney and Shetland are held at the General Register Office for Scotland (New Register House, Edinburgh). These records, along with relationship information obtained from study participants, Orkney and Shetland family history societies, and genealogies available online, were used to construct pedigrees of study participants using RootsMagic software (S&N Genealogy Supplies), which were then amended to reflect the genetic kinship between individuals using genotype data.

### Genotyping

DNA from all participants was used for genome-wide genotyping on the GSA BeadChip (Illumina) at the Regeneron Genetics Center. Monomorphic genotypes and genotypes with more than 2% of missingness and Hardy-Weinberg equilibrium (HWE) *p* < 10^−6^ were removed, as well as individuals with more than 3% of missingness. Biological sex and expected genomic sharing with first-degree relatives were used to exclude sample mix-ups.

### Exome sequencing

Quality-controlled exome sequence datasets were prepared at the Regeneron Genetics Center, following the process detailed for the UKB,^2^ with 94.6% of targeted bases having >20× coverage.

### Pipeline development

To find actionable genotypes, we first extracted all variants that map to genes in the ACMG v.3.2 list from whole-exome sequences (WESs).^1^ These variants were annotated with dbsnp v.151 using the bcftools 1.9 annotate function, using CHROM (chromosome), POS (position), ID, REF (reference), and ALT (alternate) columns. The dbsnp 151 data were downloaded from https://ftp.ncbi.nlm.nih.gov/snp/organisms/human_9606_b151_GRCh38p7/VCF/ (00-All.vcf.gz file) in March 2022. To assess their pathogenicity, we merged these variants with the ClinVar data. The variant_summary.txt.gz file of the ClinVar data was downloaded on March 18, 2024, from https://ftp.ncbi.nlm.nih.gov/pub/clinvar/tab_delimited/. Merging by CHROM, POS (genome build GRCh38), REF, and ALT alleles was necessary since rsids can be multiallelic—containing multiple variants at the same genomic position, including different nucleotide substitutions, for example, tri-allelic SNPs (A>C and A>G) and insertions or deletions (indels); therefore, merging solely by rsids might result in merging different variants.

Some of the variants present in the exomes were not identical to mutations listed in ClinVar but resulted in the same amino acid change as variants in ClinVar and thus the same consequence, commonly a frameshift with comparable functional effects. Therefore, we also merged the variants from the WESs with ClinVar data based on the change of the amino acid that the variant causes instead of the CHR, POS, REF, or ALT. Furthermore, if the following hypothetical indel occurred, TCATCTA > TCATCTCTA, then it could be named c.4insCT, c.5insTC, or c.6insCT, causing problems for merging the data using either of the two approaches. This is illustrated by the real variant rs886044536, titin (*TTN*) c.93396_93400del (GenBank: NM_001267550.2) (p.Trp31134Ter) (MIM: 188840; results). This is a deletion, GCCAAGCTAAGACT > GCCAAGACT, which can be rendered either as GCCAAG[CTAAG/^∗^]ACT or GCC[AAGCT/^∗^]AAGACT.

After merging, we next filtered the output using the ClinVar annotations to retain only pathogenic or likely pathogenic (P/LP) variants that also had a status of 1 star or higher, and an assertion of known pathogenicity (KP) and noted the mode of inheritance. Moreover, the variant class had to match the disease-causing variant classes for that gene, e.g., only loss-of-function (LoF; truncating) variants for *TTN*. Variants were also subject to manual review before being listed as potentially actionable.

### WGS

Whole-genome sequencing (WGS) data are available for 1,300 members of the ORCADES cohort^16^ and 500 members of the Viking I cohort.^17^ These datasets were used for look-ups as part of the verification process of the AV results obtained from exome sequencing.

### Sanger sequencing

Verification of the results from exome sequencing was also achieved by Sanger sequencing. A new DNA sample was picked from stored master stocks. Pre-designed primer pairs for PCR and Sanger sequencing were selected from a collection that covers over 95% of human coding exons (Primer Designer, Thermo Fisher Scientific). If validated primers were not available, then primer pairs were designed using Primer3 software (Thermo Fisher Scientific) and checked using UCSC In-Silico PCR.^18^ A standard protocol for PCR and Sanger sequencing on an Applied Biosystems 3730xl Genetic Analyzer was applied across all samples. Output files were analyzed using Sequencher DNA sequence analysis software v.5.4.6 (Gene Codes Corporation).

Validation of heterozygous short indels can be challenging with Sanger sequencing. Although still considered the gold standard, the sequencing ladders derived from, for example, the deleted and non-deleted alleles become out of synchronicity with one another (by the length of the indel in base pairs), leading to a double sequence after the indel in each direction. Illumina short-read sequencing, as used to generate the exome data, does not suffer from this problem. A further issue is that certain indels with short repeats within or near them (or in low-complexity DNA) can be rendered in multiple ways in cDNA nomenclature, e.g., *TTN* c.93396_93400del (GenBank: NM_001267550.2; p.Trp31134Ter) (see above). More common are single-base indels altering the length of homopolymers, which can be annotated as a change at any position in, for example, the poly(A) tract. Chromosome positions are affected in the same way, and so when interrogating ClinVar, we performed a secondary merge using the protein sequence nomenclature to pick up such instances.

### Participant information and clinical review of potentially actionable genotypes

The consent form and RoR participant information sheets (PISs) sent to cohort members are provided as supplemental information. Frequently asked questions about RoR are available for participants on the study website at https://viking.ed.ac.uk/for-viking-genes-volunteers/faqs/return-of-results. As stated in the consent form and PISs, a key part of the framework of our RoR approach was that all potential AVs and genotypes presented in Table S1, other than those noted as not determined, were discussed with the NHS clinical team in order to agree on the suitability for RoR to participants. Local clinical knowledge in NHS Grampian was used as part of this review process.

### EHR data linkage

This work used data provided by patients and collected by the NHS as part of their care and support, reused with permission from Public Health Scotland. NHS routine datasets linked to ORCADES and VIKING I participants in July 2021, including SMR01, the general/acute hospital inpatient and day case dataset of episode-level data, and SMR00, outpatient appointments and attendances, were accessed using a secure process, as described previously.^11^

### UKB

Frequencies for the ten drifted variants reported here were derived from the UKB WGS project and were obtained from the publicly available UKB Allele Frequency Browser (https://afb.ukbiobank.ac.uk/), which was generated by the WGS consortium under the UKB Resource (project ID 52293).

## Results

### Exome sequencing and validation

Exome sequences were generated from 4,198 participants from the Viking Genes cohort using industry-standard protocols as described in the subjects and methods. It is important to verify the exome sequence calls in the research laboratory before returning any results. We therefore attempted to validate 149 potential AV genotypes: 77 by Sanger sequencing, 48 by WGS, and 24 by both sequence analysis methods. The total of 149 includes a number of heterozygous carriers of recessive variants in genes on the ACMG v.3.2 list. 147 out of the 149 assessed were validated (98.7%). Notably, both of the variants that failed (one by Sanger sequencing alone and one by both Sanger sequencing and WGS) were only 2 kb apart in the same gene, *MSH2* (MIM: 609309). Manual review of the exome read stacks suggests that these variants are both false positives, with low read numbers and increased noise; they were not included in the total counts of AVs found.

One variant in *DES* (MIM: 125660), c.973C>T (GenBank: NM_001927.4) (p.Arg325Ter), was found to be most likely a somatic mosaic in the sample of venous blood and so was not included in our counts and not returned. In Sanger sequencing from forward and reverse strands, only a very weak T was observed, and the read counts in WGS were 19C and 1T. In 85× exome data, the total read counts (sum of forward and reverse) are 72C and 13T for a variant allele distribution of 15%.

### Pipeline for identification of potential AVs

Exome sequence data from 2,090 ORCADES participants (820 male and 1,270 female) and 2,108 VIKING I participants (843 male and 1,265 female) passed all sequence and genotype quality control thresholds (subjects and methods). The data were run through our pipeline (subjects and methods), a key component of which is the ClinVar resource (www.ncbi.nlm.nih.gov/clinvar/). ClinVar aggregates information about genomic variation and its relationship to human health and allocates a clinical significance category to each variant.^14^

In total, 59 potential AVs were observed, consisting of 27 singletons (Table S1) and 32 non-singleton variants (Tables 1 and 2). Half of the singleton variants (13/27) were observed in participants with one non-islander parent, and a further 4/27 were in volunteers with one non-islander grandparent, for a total of 63% of singletons with at least one-quarter non-Northern Isles DNA. The remaining 10 singletons presumably arose by mutation in the last few generations and are, thus, very rare or were brought into the islands by non-parental events. The variants were found in 26 different genes. For three of these genes, *BTD* (MIM: 609019), *CASQ2* (MIM: 114251), and *RPE65* (MIM: 180069), each carrier only had a single copy of a variant that acts recessively, and thus these were not actionable genotypes. In this paper, we are careful to distinguish between AVs (from the ACMG list) and actionable genotypes, the combinations of such variants that are actionable, for example, compound heterozygotes or homozygotes for autosomal recessive conditions. In total, using the 81-gene ACMG version 3.2 list, we observed actionable genotypes for 104 individuals (2.5% of the population) with 108 actionable genotypes at 39 of the variants across 23 genes. No X-linked AVs were found.

**Table 1:** Summary of non-singleton autosomal dominant actionable variants in the cohort of 4,198 participants

**Category**	**Disease**	**Gene**	**c.**	**p.**	**Clin sig**	**Actionable**	**Consequence**	**LoF**	**Stars**
Cancer	breast-ovarian cancer, familial	*BRCA1*	c.5207T>C	p.Val1736Ala	P	all P and LP	missense	0	3
		*BRCA2*	c.517-2A>G	p.?	P	all P and LP	splice acceptor	1	3
	Lynch syndrome	*MSH6*	c.3261dup	p.Phe1088fs	P	all P and LP	frameshift	1	3
	attenuated familial adenomatous polyposis	*APC*	c.154C>T	p.Gln52Ter	P	all P and LP	stop gain	1	2
	multiple endocrine neoplasia	*RET*	c.2410G>A	p.Val804Met	P/LP	all P and LP	missense	0	2
Metabolic	hereditary transthyretin amyloidosis	*TTR*	c.262A>T	p.Ile88Leu	P/LP	all P and LP	missense	0	2
Cardiovascular	long QT syndrome	*KCNH2*	c.1750G>A	p.Gly584Ser	P/LP	all P and LP	missense	0	2
		*SCN5A*	c.4716C>T	p.Gly1572 =	P/LP	all P and LP	splice donor	1	2
	dilated cardiomyopathy	*TTN*	c.1558dupA^a^	p.Thr520fs	LP	only LOF	frameshift	1	1
		*TTN*	c.67519C>T	p.Gln22507Ter	LP	only LOF	stop gain	1	1
		*TTN*	c.88837A>T	p.Lys29613Ter	P/LP	only LOF	stop gain	1	2
		*TTN*	c.93396_93400del	p.Trp31134Ter	P/LP	only LOF	frameshift	1	2
	vascular Ehlers-Danlos syndrome	*COL3A1*	c.944G>C^a^	p.Gly315Ala	LP	all P and LP	missense	0	1

The diseases are grouped into the categories of cancer, metabolic, and cardiovascular. TTN c.1558dupA does not appear in ClinVar, so the star status and clinical significance are given for a variant causing the identical amino acid change. c., coding sequence change; p., protein sequence change; Clin sig, clinical significance according to ClinVar; LP, likely pathogenic; P/LP, pathogenic/likely pathogenic; actionable, variant categories considered to be actionable; LoF, loss of function; consequence, the molecular consequence of the variant; star, ClinVar star status.

a These variants were not considered actionable by the NHS Clinical Genetics team and were not returned.

**Table 2:** Summary of non-singleton autosomal recessive actionable variants in the cohort of 4,198 participants

**Category**	**Disease**	**Gene**	**c.**	**p.**	**Clin sig**	**Actionable**	**Consequence**	**LoF**	**Stars**
Cancer	MYH-associated polyposis	*MUTYH*	c.452A>G	p.Tyr151Cys	P/LP	P and LP (2 var)	missense	0	2
		*MUTYH*	c.1103G>A	p.Gly368Asp	P/LP	P and LP (2 var)	missense	0	2
		*MUTYH*	c.1130C>T	p.Pro377Leu	P	P and LP (2 var)	missense	0	2
Metabolic	Wilson disease	*ATP7B*	c.122A>G	p.Asn41Ser	P/LP	P and LP (2 var)	missense	0	2
		*ATP7B*	c.1745_1746del	p.Ile582fs	P/LP	P and LP (2 var)	frameshift	1	2
		*ATP7B*	c.1772G>A	p.Gly591Asp	P/LP	P and LP (2 var)	missense	0	2
		*ATP7B*	c.2605G>A	p.Gly869Arg	P/LP	P and LP (2 var)	missense	0	2
		*ATP7B*	c.2662A>C	p.Thr888Pro	P/LP	P and LP (2 var)	missense	0	2
		*ATP7B*	c.2930C>T	p.Thr977Met	P	P and LP (2 var)	missense	0	2
		*ATP7B*	c.3007G>A	p.Ala1003Thr	P/LP	P and LP (2 var)	missense	0	2
		*ATP7B*	c.3083_3085delinsG	p.Lys1028fs	P	P and LP (2 var)	frameshift	1	2
		*ATP7B*	c.3207C>A	p.His1069Gln	P	P and LP (2 var)	missense	0	2
	biotinidase deficiency	*BTD*	c.1308A>C	p.Gln436His	P/LP	P and LP (2 var)	missense	0	2
		*BTD*	c.1552C>T	p.Arg518Cys	P	P and LP (2 var)	missense	0	2
	hereditary hemochromatosis	*HFE*	c.845G>A	p.Cys282Tyr	P	C282Y homs only	missense	0	2
	Pompe disease	*GAA*	c.841C>T	p.Arg281Trp	LP	P and LP (2 var)	missense	0	3
		*GAA*	c.1194+3G>C	p.?	LP	P and LP (2 var)	splice region	0	3
		*GAA*	c.1210G>A	p.Asp404Asn	P	P and LP (2 var)	missense	0	3
		*GAA*	c.2238G>C	p.Trp746Cys	P	P and LP (2 var)	missense	0	3

The diseases are grouped into the categories of cancer and metabolic. c., coding sequence change; p., protein sequence change; Clin sig, clinical significance according to ClinVar; LP, likely pathogenic; P/LP, pathogenic/likely pathogenic; actionable, variant categories considered to be actionable; LoF, loss of function; consequence, the molecular consequence of the variant; star, ClinVar star status; 2 var, 2 variants must be present, so either homozygous or compound heterozygous for the P/LP variant; homs only, only homozygotes for the named variant should be reported.

The level of support for each review status in ClinVar decreases from four stars downwards (https://www.ncbi.nlm.nih.gov/clinvar/docs/review_status). The great majority of the AVs we found were 3 (reviewed by an expert panel) or 2 (criteria provided, multiple submitters, no conflicts) stars (Table S1). Three of the non-singleton autosomal dominant AVs (Table 1) had a review status in ClinVar of 1 star, all of which had a single submitter. The paucity of submissions often indicates the rarity of the variant, which in turn makes assessment of pathogenicity, and hence actionability, more challenging.

### Actionable genotype co-occurrences

We identified four individuals with actionable genotypes in two separate genes (Table 3). We expect some such co-occurrences because of independent assortment, and we should see these according to the product of the allele frequencies of the variants in question. If one or both have drifted upwards in frequency, then more will be seen. We found that two of these four Viking Genes individuals had AVs in two different cancer susceptibility genes, while two had actionable genotypes in a cancer susceptibility gene and a metabolic gene.

**Table 3:** Actionable genotype co-occurrences

**Gene 1**	**Variant ID**	**Inheritance**	**Genotype**	**Gene 2**	**Variant ID**	**Inheritance**	**Genotype**
*BRCA1*	c.5207T>C	AD	het	*PMS2*	c.137G>T	AD	het
*BRCA2*	c.6275_6276del	AD	het	*MSH6*	c.742C>T	AD	het
*BRCA2*	c.517-2A>G	AD	het	*GAA*	c.841C>T	AR	hom
*BRCA2*	c.5073dupA	AD	het	*HFE*	c.845G>A	AR	hom

AD, autosomal dominant; AR; autosomal recessive; het, heterozygous; hom, homozygous.

### Drifted AVs in Northern Isles populations

The isolated Northern Isles populations carry different sets of rare variants compared to cosmopolitan populations. In total, 10 AVs across 7 genes (*BRCA1* [MIM: 113705], *BRCA2* [MIM: 600185], *ATP7B* [MIM: 606882], *TTN* [MIM: 188840], *KCNH2* [MIM: 152427], *MUTYH* [MIM: 604933], and *GAA* [MIM: 606800]) have risen in frequency by at least 50-fold through the action of genetic drift in the Northern Isles (Table 4). These include results published previously on a *BRCA1* breast and ovarian cancer predisposition variant that is 470-fold more common in Orkney than in the UKB,^11^ a *KCNH2* long QT syndrome variant that is ∼90-fold more common in Shetland,^10^ and a P *BRCA2* variant ∼155-fold more common in Shetland.^12^

**Table 4:** Founder effects on ten actionable variants in Orkney and Shetland

**Gene**	**c.**	**p.**	**Disease**	**inher**	**maf.ork**	**ork/ukb**	**maf.shet**	**shet/ukb**	**Group**	**Origin**
*BRCA1*	c.5207T>C	p.Val1736Ala	hereditary breast ovarian cancer	AD	0.00478	470	0	0	Orkney	Westray
*BRCA2*	c.517-2A>G	p.?	hereditary breast ovarian cancer	AD	0	0	0.00237	155	Shetland	Whalsay
*ATP7B*	c.3083_3085delinsG	p.Lys1028fs	Wilson disease	AR	0	0	0.00475	180	Shetland	Burra
*ATP7B*	c.3007G>A	p.Ala1003Thr	Wilson disease	AR	0	0	0.00356	350	Shetland	Sandsting/Lunnasting/Delting
*ATP7B*	c.1772G>A	p.Gly591Asp	Wilson disease	AR	0.00215	66	0	0	Orkney	Westray
*TTN*	c.93396_93400del	p.Trp 31134Ter	dilated cardiomyopathy	AD	0	0	0.00380	3700	Shetland	Yell
*TTN*	c.67519C>T	p.Gln22507Ter	dilated cardiomyopathy	AD	0.00048	700	0.00071	470	Orkney	West Mainland
*KCNH2*	c.1750G>A	p.Gly584Ser	long QT syndrome	AD	0	0	0.00119	90	Shetland	Aithsting, Unst
*MUTYH*	c.1205C>T	p.Pro377Leu	*MYH*-associated polyposis	AR	0.00431	70	0.00047	8	Orkney	Westray
*GAA*	c.1210G>A	p.Asp404Asn	Pompe disease	AR	0.00287	74	0	0	Orkney	St. Andrews

Drifted variants were defined as actionable variants with an increase in frequency over 50-fold higher than the UKB, a pedigree origin before c.1800 CE and a minor-allele count of ≥5. For the UKB, we used the allele frequency browser (based on ∼490,000 WGS). c., complementary DNA position and nucleotide change; p., protein position and amino acid change; inher, inheritance; maf.ork, minor-allele frequency in Orkney; ork/ukb, fold uplift in frequency in Orkney over the UKB; maf.shet, minor-allele frequency in Shetland; shet/ukb, fold uplift in frequency in Shetland over the UKB; group, island group the variant has drifted upwards in; origin, sub-population (parish, isle, or region) where the variant arose (by tracing back pedigrees) or has become particularly common.

Most AVs in the ACMG list have a dominant mode of inheritance and so are actionable if heterozygous, but some are only considered actionable if present in two copies. Table 4 includes five drifted variants in Northern Isles populations that act recessively (also listed in Table 2). These are in *ATP7B* (three drifted variants segregating in our cohorts), *MUTYH*, and *GAA*. Wilson disease (MIM: 277900) is a disorder of copper metabolism caused by variants in the copper-transporting ATPase gene *ATP7B*. The marked discrepancy between the “genetic prevalence” in populations and the number of clinically diagnosed individuals with Wilson disease is probably due to both reduced penetrance of many *ATP7B* mutations and failure to diagnose individuals with this highly treatable disorder.^19^ We identified three founder effects on recessive *ATP7B* Wilson disease variants, two of them in Shetland, which gives rise to a substantial risk of homozygosity or compound heterozygosity. The combined allele frequency in Shetlanders is 0.8%. Together with five other alleles segregating at lower frequencies, the sum P/LP allele frequency is 1.12%; thus, about 1/45 Shetlanders are carriers of P/LP *ATP7B* variants. Under random mating, this predicts that about 1/8,000 will be homozygotes or compound heterozygotes and at risk of being affected. Indeed, one homozygote for the drifted variant c.3007G>A (p.Ala1003Thr) was found.

There is a founder effect on a recessive *MUTYH* variant (c.1130C>T [GenBank: NM_012222.2] [p.Pro377Leu]) in Orkney (0.86% carriers, 70× more common than the UKB), causing *MYH*-associated polyposis (MIM: 608456) (Table 4). While not increased in frequency compared to other UK populations, there are two further relatively common *MUTYH* variants segregating in the Orcadian population, bringing the combined carrier frequency to 2.9%. This suggests that 1/35 Orcadians are carriers and, assuming random mating among Orcadians, an expectation that ∼1/5,000 will be at risk of *MYH*-associated polyposis. Consistent with this, we did observe a compound heterozygote in the Orkney data.

Finally, there are also founder effects in Shetland on two dominant LoF *TTN* variants, increasing the risk of dilated cardiomyopathy (DCM; MIM: 604145) (Table 4). One of these, c.93396_93400del (GenBank: NM_001267550.2) (p.Trp31134Ter), is > 3,000-fold more common in Shetland than in the UKB, where there is only a single instance in 917,682 alleles. Together with a third returnable *TTN* variant that does not meet our thresholds to be defined as drifted, the combined allele frequency is 0.5%, meaning that 1/100 Shetlanders have an actionable *TTN* genotype. In total, we observed 24 people with *TTN* LoF variants (23 of which are accounted for by these three alleles).

### An ultra-rare drifted TTN variant

The penetrance of the *TTN* alleles included in ClinVar as P or LP is variable and age related. However, the deletion of 5 nucleotides, c.93396_93400del (GenBank: NM_001267550.2), causes a frameshift, which creates a premature translational stop signal (p.Trp31134Ter), predicted to result in an absent or disrupted protein product. This variant is in the A-band of *TTN*. Truncating variants in the A-band of *TTN* are significantly overrepresented in individuals with DCM and are considered to be LP for the disease.^20^ TTN truncating variants (TTNtvs) located in exons highly expressed in the heart (proportion spliced in [PSI] > 0.9, or hiPSI), such as this one, increase the odds of DCM by 11- to 19-fold.^21^ This variant has, therefore, been classified as LP in ClinVar. We examined data from 347 individuals ascertained with DCM and found to carry hiPSI TTNtvs in DCM cohort studies from Europe, the USA, and Australia and also interrogated data from TTNtv individuals in the UKB,^22^ the Geisinger MyCode Biobank, and the Penn Medicine Biobank (168 affected with DCM and 2,409 unaffected).^21^ In total, we observed 1,200 distinct hiPSI TTNtvs in 2,094 individuals. The c.93396_93400del (GenBank: NM_001267550.2) (p.Trp31134Ter) variant was observed twice (1 in the UKB and 1 in MyCode), both times in individuals without a diagnosis of DCM. The variant was not observed in the 100,000 Genomes Project^23^ (670 DCM participants, 62,330 non-DCM participants) or All of Us^24^ (245,460 total participants, of whom 125,860 were reported as European heritage) and, therefore, can be considered ultra-rare.

Analysis of linked data from the electronic health records (EHRs) for cardiomyopathy (ICD-10 I42), atrial fibrillation (ICD-10 I48), or heart failure (ICD-10 I50) shows that the 16 people with the drifted *TTN* variant c.93396_93400del (GenBank: NM_001267550.2; p.Trp31134Ter) we identified in VIKING I are nearly four times more likely (*p* = 0.02, Fisher’s exact test) to have an inpatient or day patient record for cardiomyopathy (1/16), atrial fibrillation (2/16), or heart failure (2/16) than people with no actionable *TTN* variant. A total of 4 of the 16 heterozygotes have entries for at least one of these conditions, i.e., a nominal combined penetrance of 25% versus a prevalence of 6.8% in 4,133 individuals without *TTN* LoF variants. Analysis of ECG data collected in the VIKING 1 recruitment clinic from 15 of the people with the drifted variant also shows an overrepresentation of features such as first-degree atrioventricular block (3/15) or right bundle branch block (4/15), affecting a total of 7 individuals. In one Shetland *TTN* c.93396_93400del (GenBank: NM_001267550.2) (p.Trp31134Ter) family investigated clinically, the condition has typically been detectable on echocardiography around age 30, but symptoms have not developed until later. One individual required a cardiac resynchronization therapy defibrillator, while another is asymptomatic despite an abnormal echocardiogram in their late 50s. Together with the 7 entries in ClinVar submitted to date, we now report this further direct evidence for the pathogenicity of this ultra-rare drifted *TTN* variant.

### Impact of sub-population gene pools

The drifted variants are not shared between the two archipelagos except for *TTN* c.67519C>T (GenBank: NM_001267550.2) (p.Gln22507Ter), which is observed in Shetland among individuals with recent Orcadian ancestry, and *MUTYH* c.1130C>T (GenBank: NM_012222.2) (p.Pro377Leu), for which the Shetland individuals have no recent Orcadian ancestry. This latter variant may have been introduced to the Northern Isles more than once, or there may be unrecorded, non-parental events connecting the Orcadian and Shetlandic kindreds.

**Figure 1:**
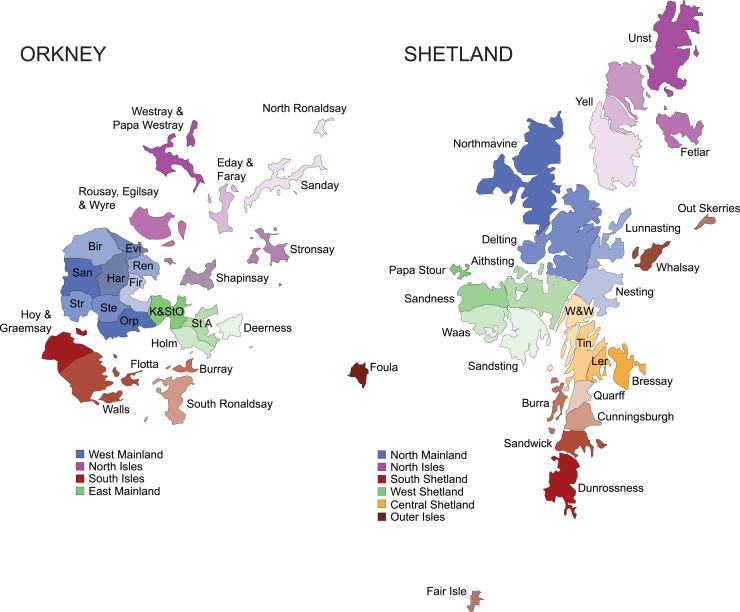
Isles and parishes of Orkney and Shetland Each archipelago consists of a larger island called the Mainland, with surrounding smaller isles. Both island groups can be divided into 4–6 larger areas, such as West Shetland (known locally as the West side) or the West Mainland of Orkney, which in turn are organized into 25 parishes or isles. Founder effects are usually concentrated in one parish/isle or in one of the larger contiguous areas. W&W, Whiteness & Weisdale; Tin, Tingwall; Ler, Lerwick; St A, St. Andrews; K&StO, Kirkwall & St. Ola; Orp, Orphir; Ste, Stenness; Str, Stromness; San, Sandwick (Orkney); Bir, Birsay; Evi, Evie; Ren, Rendall; Fir, Firth; Har, Harray.

The great majority of these AVs can be traced back to individual isles within Orkney or Shetland or, in some cases, individual parishes: 13–15 ancient geographic, legal, and religious subdivisions of the Mainlands of Orkney and Shetland (Figure 1). Among published variants, 95% of people with the drifted *BRCA1* variant traced their genealogies to Westray, in the North Isles of Orkney,^11^ while 78% of those with the drifted *BRCA2* variant trace back to Whalsay, an isle in Shetland.^12^ Likewise, people with the drifted *KCNH2* variant from Viking Genes and NHS Clinical Genetics^10^ trace back to either the parish of Aithsting in the West Mainland of Shetland or Unst in the North Isles of Shetland (Figure 1; Table 4).

Among the drifted variants reported here, 80% of the carriers of the most drifted *ATP7B* variant have genealogies going back to Burra Isle, Shetland. There is also a strong founder effect on the most highly drifted *TTN* variant, c.93396_93400del (GenBank: NM_001267550.2) (p.Trp31134Ter), this time in Yell, in the North Isles of Shetland, with all those with the variant tracing their family trees back there. Further fine-scale, within-archipelago population structuring of clinically important variants is observed in Westray, in the North Isles of Orkney, with another *ATP7B* variant having drifted to high frequencies there and all heterozygotes tracing pedigrees there, similar to a *MUTYH* variant, where 60% of people have Westray genealogies; indeed, we observed a compound heterozygote with ancestry from that isle.

### Genetic down drift

It is more difficult to quantify which variants have drifted down in frequency, but the near absence of familial hypercholesterolemia (FH) (MIM: 143890 and MIM: 144010) variants is notable, with only one actionable genotype across >4,000 subjects. This *LDLR* (MIM: 606945) variant was observed in Shetland and is the known c.551G>A (GenBank: NM_000527.5) (p.Cys184Tyr) West of Scotland founder variant^25^; it may have been brought to the isles by recent gene flow from Mainland Scotland. No FH variants were observed in Orkney. No P/LP *APOB* (MIM: 107730) or *PCSK9* (MIM: 607786) variants were observed. Significantly fewer volunteers from the Northern Isles carried FH variants than observed in European ancestry participants from the UKB (*p* < 0.0001, Fisher’s exact test). The frequency in the Northern Isles (1/4,198) is more than 10-fold lower than that in UKB European ancestry subjects (1/288).^26^

### The RoR process

**Figure 2:**
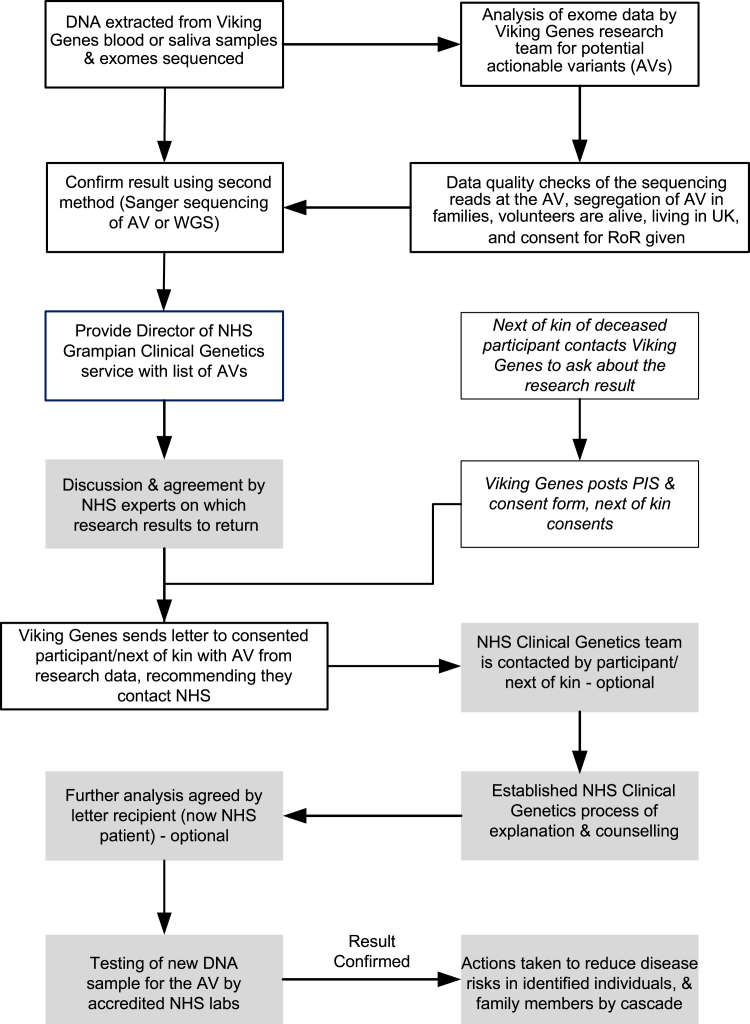
Overview of Viking Genes return of results Processes implemented by the Viking Genes research team are boxed with no shading; processes implemented by the NHS clinical team are shaded. PIS, participant information sheet (see supplemental information).

With reference to the Association of Clinical Genomic Science (ACGS) guidelines (https://www.acgs.uk.com/media/12533/uk-practice-guidelines-for-variant-classification-v12-2024.pdf) and local clinical knowledge, four variants in a total of 10 people with actionable genotypes from our pipeline were not deemed returnable by the NHS clinical team (Tables 5 and S1). Our next goal was to implement a process of RoR to participants, which is summarized in Figure 2. Key principles include the use of informed consent, respecting the right not to know, checking the quality of the research results as far as possible before sharing a potential AV ID, decisions about whether to return a variant result taken by NHS clinical experts, counseling provided through the NHS, and verification of all the research results using a newly collected sample in an accredited clinical laboratory.

**Table 5:** Summary of people with actionable genotypes and results returned

**People with actionable genotypes**	**Category**
64	results letters sent, including one to next of kin
33	not consented – did not reply to return of results consent invitation
8	deceased and no request from next of kin
2	answered no to return of results consent invitation
10	not deemed returnable by NHS
117	total people with actionable genotypes, including *BRCA1* males and accounting for co-occurrences

Consent was only sought 8–18 years after recruitment, which is probably why 28% of people with actionable genotypes did not reply to the invitation. A further 11% were deceased, while 2.7% replied and chose not to have return of results. NHS, National Health Service.

We obtained a favorable opinion from the research ethics committee to return results of genotypes that have the potential to improve the health of the individuals carrying them. We subsequently obtained further permission to contact males with P *BRCA1* variants. This was justified by the fact that although there is no clinical action to be taken with respect to the letter recipient, the information could be of value to their female relatives, who may not be Viking Genes research participants. There are 13 males with the *BRCA1* c.5207T>C (GenBank: NM_007294.4) p.Val1736Ala) variant in the ORCADES cohort^11^ and none in VIKING I, so the addition of these participants would increase the total number of actionable genotypes from 108 to 121. Since there are four individuals with two actionable genotypes each (Table 3), this corresponds to a total of 117 people with actionable genotypes including *BRCA1* males and accounting for co-occurrences (Table 5).

We further obtained permission from the research ethics committee to offer actionable results to the next of kin of deceased participants if we found any that might have been relevant to the health of the participant who died, and therefore potentially their family, during our research. Prior to submitting this amendment, we surveyed the Viking Genes public-participant involvement (PPI) panel. They were unanimously positive, answering "yes" to the question, "Would you support the offer of consent to next of kin, to allow them to obtain results about AVs their family member might have carried?" To date, six next of kin have provided their consent to receive this information, with one actionable genotype disclosed. This option is shown schematically in Figure 2.

The process we have implemented is in good accordance with a checklist of components for RoR developed by an interdisciplinary panel of European experts.^27^ In Viking Genes, the return of medically actionable genetic results to participants is optional, i.e., an opt-in approach. This has legal, philosophical, and ethical foundations to allow individual autonomy and the right not to know. This is achieved at three separate points in the process of implementation (Figure 2). Letters containing results are only sent with the consent of the recipient. Upon receipt, the participant can decline any further investigation by not contacting the NHS about the research finding. Finally, after genetic counseling, the individual can decide not to have the research result confirmed in a clinical setting. It should also be noted that a participant can get in touch with Viking Genes at any time to request to change the consent they gave about RoR, in either direction, or to withdraw from the study. The research team communicates with Viking Genes volunteers through regular newsletters and social media and invites them to complete occasional research surveys. A subset of participants in the study forms the PPI group. Therefore, we aim to ensure both that understanding of the opportunity to receive results is high and that the processes we are implementing have the support of the study participants and the populations they are drawn from.

### Initial outcomes from issuing RoR letters

The text of the letters alerting the volunteer to the research result is written in lay language, agreed on by the research and NHS teams, and the template document was given a favorable opinion by the research ethics committee. The NHS clinic team is not made aware of the identity of the research participants but approves the return of the variants, which are also noted in the letters. Letters were not sent to all eligible participants at one time. Instead, the 64 RoR letters issued (Table 5) were posted over the course of almost exactly 1 year. This was designed to prevent the NHS clinics from being overwhelmed but also means that outcomes are still being accumulated. For example, it is too early to tally the number of family members contacted by cascade testing from each index case. In the NHS, each index case leads to, on average, three subsequent cascade tests, half of which are positive.^28^ In the case of the Shetland *BRCA2* founder variant c.517-2A>G (GenBank: NM_000059.4) (p.?), a total of 56 people were eligible for cascade letters from just 5 consented volunteers with the variant in Viking Genes, suggesting a higher level of knowledge of family structure among our participants. We observe that RoR letter recipients take a variable length of time to decide whether to contact the NHS, and not all choose to do so.

In all three of the previously reported autosomal dominant actionable founder variants,^10^^,^^11^^,^^12^ we identified heterozygotes in our research populations who could not have been ascertained from oral-history-based cascade testing. Likewise, many of the index cases described here were found to be in family groups not previously recorded by the clinical geneticists. Out-patient NHS EHR data showed that only 9% of our people with actionable genotypes had interacted with clinical genetics, compared with 3.6% of the remainder of the cohorts, a 2.5-fold increase (*p* < 0.002, χ^2^). This provides further evidence that a substantial number of the research participants who received a RoR letter were previously unaware of the genomic medicine finding and now have the opportunity for preventative medical intervention as a result of the Viking Genes RoR program.

## Discussion

### Survey of AVs

We observed actionable genotypes for 39 autosomal AVs, 34 dominant and 5 recessive, and no X-linked variants. Ten founder variants showed strong enrichment, having risen >50-fold in frequency in Orkney or Shetland, while otherwise commonly seen variants, notably those causing FH, were at least 10-fold lower in frequency. Importantly, both *TTN* and *ATP7B* contain multiple drifted variants, which sum together to even higher frequencies of (L)P variation.

The ten AVs that have drifted up to much higher frequencies in Orkney or Shetland than in the general UK population mostly have a clear origin in a particular parish or isle within the Northern Isles 200 or more years ago, highlighting the continuing clinical importance of these historic sub-population gene pools. As is the case with other genetic isolate populations,^29^ the combination of multiple founder variants can result in a significant summed population frequency, sometimes in conjunction with non-drifted variants. At the same time, other variants have drifted toward loss, balancing the genetic burden of disease risk.

### Comparisons with other populations surveyed for AVs

Overall, these two UK isolate populations have similar percentages of actionable genotypes (2.5%) compared to cosmopolitan populations worldwide. However, the number with one or more actionable genotypes depends on the gene list, rules applied (including bespoke curation), and date of analysis. The ACMG list of recommendations of genes and phenotypes is updated annually, whereas ClinVar is designed to enable the ongoing evolution and development of knowledge regarding variations and associated phenotypes, and its website is updated weekly. The status of a variant, particularly if rarely observed worldwide, may, therefore, change over time as more data are submitted, interpreted, and reported in ClinVar. This makes it challenging to compare our Viking Genes actionable genotype percentage with populations reported in other publications. In the first 50,000 exome sequences from the UKB population cohort, an actionable genotype figure of 2.0% was reported.^2^ However, that analysis used v.2.0 of the ACMG list,^30^ which contained 59 genes rather than the 81 in v.3.2 that were surveyed here. Similarly, a figure of 2.8% can be calculated from the DiscovEHR study^31^ and 2.3% in Iceland^32^ using ACMG list v2.0. Another way to compare with studies in other populations is to use the ACMG 2.0 (59 gene) list to assess our data in the Northern Isles population, in which case the percentage with actionable genotypes drops to 1.4%.

The inclusion of *HFE* (MIM: 613609) c.845G>A (GenBank: NM_000410.3) (p.Cys282Tyr) homozygotes, in later versions of the list, would, on its own, increase the percentage of actionable genotype individuals in the UKB from 2.0% to 2.6%. This is based on the *HFE* variant having a reported homozygous frequency of 0.6%, or 1 in 156, in the UKB.^33^ We, therefore, show here that the Northern Isles populations have an AV percentage that is in a similar range to the rest of the population of the UK as sampled in the UKB; to the DiscovEHR study of people in Pennsylvania, USA; and to the Icelandic population (4.0% in the most recent analysis, including manual curation)^32^ but much lower than that reported for the Old Order Amish of Pennsylvania.^34^

We identified a sufficient number of individuals to provide specific evidence for pathogenicity of one drifted variant in *TTN*, c.93396_93400del (GenBank: NM_001267550.2) (p.Trp31134Ter). A total of 44 genes were recently asserted to be implicated in non-syndromic DCM by the ClinGen DCM gene curation expert panel.^35^ *TTN* is one of 11 genes with definite evidence and featured in a gene-based analysis of penetrance and clinical phenotype in 18,665 UKB participants.^36^ A sub-analysis for genes including *TTN* revealed DCM or early DCM features in 45.4% of individuals.^36^ Moreover, significant excess mortality was observed among people with TTNtvs in Dutch founder pedigrees, driven by subjects ≥60 years.^37^

### RoR to research volunteers: Benefits and challenges

The RoR processes that we successfully implemented for Viking Genes participants as described here were somewhat more straightforward than they would be in cosmopolitan populations due to the reduced number of different AVs in the ancestral populations of the Northern Isles of Scotland and the high support for and trust in scientific research in these communities. Nonetheless, the processes and experiences of returning actionable genetic results in Viking Genes are directly relevant as an exemplar (especially in the UK) for other research cohorts interested in the feasibility and logistics of the return of actionable results to their participants. Our recent recruitment to a new cohort study, VIKING II, was eligible to people of Northern Isles ancestry regardless of domicile.^15^ At the outset of recruitment, VIKING II offered the option of consent to return of selected clinically actionable results. This option was chosen by an overwhelming majority (98%), 6,054 of the 6,178 participants who consented and completed the study questionnaire. This is in good agreement with several other studies that also show strong support for receiving actionable findings, both in theory and in practice.^38^^,^^39^ These views may mean that the return of actionable genetic findings from research will, in the future, become the default practice.^39^ However, the costs, harms, and benefits of returning actionable results are still at an early stage of being fully characterized.

For some diseases in the ACMG list, there can often be a long delay before accurate diagnosis due to the overlap of symptoms with other more prevalent conditions. Examples we have found in Viking Genes include Pompe disease (MIM: 232300), caused by a deficiency of the enzyme acid alpha-glucosidase (GAA), which can lead to nerve and muscle problems; Wilson disease (MIM: 277900), caused by copper overload; and the iron-overload condition hereditary hemochromatosis (MIM: 235200) (*HFE*). Early diagnosis and, thus, early intervention can have an impact on future quality of life. Furthermore, in addition to the medical intervention available for each actionable result, a clearer diagnosis/etiology following the RoR can also be of value for those already affected, particularly for conditions like cardiomyopathy, which has complex symptoms and presentation. Increased awareness of these otherwise rare conditions among healthcare workers in the Northern Isles could lead to decreased times to diagnosis, with concomitant benefits to patients.

Cross-sectional approaches, for example, to understand the penetrance of rare variants in cardiomyopathy-associated genes, can provide valuable data,^40^ and genome-first evaluation at scale can enable new diagnoses missing from clinical healthcare.^41^ However, the scientific literature is only just beginning to provide insight into how the ACMG guidelines have been translated into precision health outcomes for the recipients of the findings: for some genes, benefits clearly outweigh harms, while for others, there is little evidence as of yet.^42^^,^^43^^,^^44^ This is likely to gather pace in the near future as more large-scale initiatives, such as All of Us,^3^ the Healthy Oregon Project,^45^ and the Colorado Center for Personalized Medicine,^46^ implement the return of actionable results. Furthermore, disease manifestation, healthcare outcomes, and costs of disclosure have recently been described for actionable findings in a UK setting from the Genomics England 100,000 Genomes Project.^47^ Genomics England returned results for actionable genotypes in 13 genes within the 81 genes on the ACMG v.3.2 list for hereditary cancer syndromes and FH, plus carrier status for cystic fibrosis (MIM: 219700; not on the ACMG list). Due to a lack of consensus on benefit versus harm and the challenges of reduced penetrance, Genomics England chose not to inform people with actionable genotypes, increasing the risk of long QT syndrome, cardiomyopathies, aortopathies, further inherited cancers, metabolic diseases, and the most frequent genetic disease, hereditary hemochromatosis (www.genomicsengland.co.uk/initiatives/100000-genomes-project/additional-findings).

Jensson et al.^32^ found shorter median survival among persons with actionable genotypes than among noncarriers in Iceland. Specifically, they report that having an actionable genotype in a cancer gene on the ACMG list was associated with a lifespan that was 3 years shorter than that among noncarriers, with causes of death among those with actionable genotypes attributed primarily to cancer-related conditions. Furthermore, while many studies have demonstrated the diagnostic and therapeutic value of exome sequencing or WGS in critically ill children, a recent study shows that the diagnostic utility of exome sequencing in critically ill adults is similar, largely due to uncovering variants in medically actionable genes.^48^ These recent studies strengthen the justification for RoR to research participants.

Our data highlight the clinical relevance of local genetic sub-populations, as most of the founder variants are also associated with one or another parish or isle, with deep genealogies linking most participants with the variants to ancestors from there (notwithstanding ancestral non-parental events, which obscure the connection between genetics and geography). Within each archipelago, it is natural that historic barriers to marriage in the form of storm-prone, strongly tidal ocean and sea channels and sounds have given rise to sub-population gene pools or micro-isolates.^7^^,^^49^ Within the main islands, the parishes are sometimes separated by low hills or sea firths, and sometimes not, but marriage records show they were demographically important over the centuries.

### Population screening opportunities

Population screening in a clinical context is currently available in some populations that show founder effects, such as an NHS program offering *BRCA* testing to those with Jewish ancestry (https://www.nhsjewishbrcaprogramme.org.uk/). Charitable organizations are also increasingly making specific genetic tests available to populations of higher risk. Examples include improving the prevention and diagnosis of Jewish genetic disorders in the UK (https://www.jnetics.org/) and the provision of postal tests for genetic hemochromatosis (https://www.haemochromatosis.org.uk/).

Our estimates of the frequency of the *BRCA1* and *BRCA2* AVs in Westray and Whalsay, respectively (1/19 and 1/43),^12^ show that the local gene pool frequencies can be much higher than the archipelago-wide frequencies. This in turn suggests that individuals with ancestry from islands such as Yell, Burra, and Westray are at a higher risk than the Shetland- or Orkney-wide data suggest. The ability to target testing to those with an origin in a particular parish may improve the cost effectiveness of future screening. At the same time, with increasing movement, younger generations and inhabitants of the towns of Kirkwall and Lerwick (and, of course, the diasporas outside the Northern Isles) tend to have more mixed ancestry, so the importance of these micro-isolates is waning.

We report four actionable genotype co-occurrences. Many more instances in other populations are likely to be uncovered in the future, as genetic analyses move from limited gene panels toward WESs and WGS. A recent review, for example, describes multi-locus inherited neoplasia allele syndrome (MINAS), which refers to individuals with germline P variants in two or more cancer susceptibility genes.^50^

We have not investigated LoF variants in genes in which LoF is a known cause of disease that may be present in our data but which are not reported in ClinVar. There may be potentially important ultra-rare variants in this class of expected P variants, which warrants future research. Furthermore, clinically relevant variants that lie outside of exomes and structural variants may be uncovered in subsequent research. However, except for the most drifted variants, the assessment of pathogenicity will always be limited by the modest numbers of instances likely to be found in our cohorts.

We also note that the high levels of engagement and knowledge of kinship led to a higher uptake of cascade testing, and thus a higher-than-expected demand on clinical services, compared to previous cascade testing statistics reported in the NHS.^28^

Studies of population genetic structure using unselected genome-wide markers^7^^,^^51^ are not always considered relevant for clinical genetics. However, a number of reports now demonstrate the consequences of such population structure for clinical genetics at the population level. A case in point is the Irish Travellers, shown to be a distinct population isolate of Irish origin^52^ with their own suite of Mendelian conditions at high frequencies.^53^ Similarly, the genetic isolation of Orkney and Shetland has been shown to translate into clinically important differences at the population level, from the earliest studies of Northern Isles genetics showing a founder effect on the *SOD1* (MIM: 147450) c.272A>C (GenBank: NM_000454.5) (p.Asp91Ala) allele^54^ to novel Mendelian conditions^55^^,^^56^^,^^57^ and distinct blood group/isozyme^58^ and uniparental^6^ markers. More recently, numerous founder effects, both on Mendelian recessive alleles^8^ and on dominant AVs^10^^,^^11^^,^^12^ have been described.

Orcadians and Shetlanders thus belong to a group of European heritage populations, including Ashkenazi Jews and Irish Travellers, where high frequencies of important clinical variants have arisen from genetic drift, with concomitant simplification of the gene pools providing valuable opportunities for population-wide, cost-effective targeted genetic screening.

## Data and code availability

There is neither research ethics committee approval nor consent from Viking Genes participants to permit open release of the individual-level research data underlying this study. The datasets generated and analyzed during the current study are, therefore, not publicly available. Instead, the research data and/or DNA samples are available from accessQTL@ed.ac.uk upon reasonable request following approval by the data access committee and in line with the consent given by participants. The ClinVar-exome pipeline code is available on GitHub (https://github.com/viking-genes/clinvar_pipeline).

## Acknowledgments

This work was funded by the 10.13039/501100000265Medical Research Council (MRC) University Unit award to the MRC Human Genetics Unit, University of Edinburgh; MC_UU_00007/10; and a 10.13039/100010269Wellcome Trust Institutional Translational Partnership Award (University of Edinburgh 222060/Z/20/Z-PIII031). L.K. was supported by an RCUK Innovation Fellowship from the National Productivity Investment Fund (MR/R026408/1). ORCADES was supported by the Chief Scientist Office of the 10.13039/100012095Scottish government (CZB/4/276 and CZB/4/710), a Royal Society URF to J.F.W., and Arthritis Research UK. J.S.W. was supported by the MRC (UK), the Sir Jules Thorn Charitable Trust (21JTA), the 10.13039/501100000274British Heart Foundation (RE/18/4/34215), and the NIHR Imperial College Biomedical Research Center. This research has been conducted using the UKB Resource under application nos. 47602 and 19655. Emily Weiss and Reka Nagy assembled the Orkney pedigree, and Barbara Gray the Shetland pedigree, using records at the General Register Office, the Shetland Family History Society, and study information, building on earlier pedigree work by Ruth McQuillan and Jim Wilson. Sanger sequencing was performed by the technical services team at the MRC Human Genetics Unit. We thank the NHS Grampian clinical genetics team and the Viking Genes research team (in particular David Buchanan, Rachel Edwards, and Craig Sinclair) for their contributions to the implementation of the RoR process. Finally, we thank the people of the Northern and Western Isles for their involvement in and ongoing support of our research.

## Author contributions

S.M.K. managed the project and drafted the manuscript. L.K. created and implemented the ClinVar-exome pipeline, with input and support from K.J., and analyzed the exome datasets. M.D.M. updated the pipeline. C.D. validated exome data by PCR and Sanger sequencing of genomic DNA. M.H. validated exome data by analysis of WGS data. S.M.K., Z.M., and J.F.W. planned the RoR methodology. Z.M. and J.D. provided information on the clinical epidemiology of the Northern Isles and clinically reviewed the variants to be reported. J.S.W., S.L.Z., and P.K.T. interpreted phenotype data in individuals with cardiomyopathy variants. G.T. and A.R.S. conceived and managed the Viking Genes exome sequencing. Z.M. led the NHS RoR clinical team, which included genetic counselors E.C. and L.S., who discussed actionable results with participants. J.F.W. is the chief investigator of Viking Genes, was awarded funding to implement the work, analyzed the data, and helped draft the manuscript. All authors provided input and feedback on drafts of the manuscript. For the purpose of open access, the author has applied a Creative Commons Attribution (CC BY) license to any author-accepted manuscript version arising from this submission.

## Declaration of interests

A.R.S. and G.T. are employees and/or stockholders of Regeneron Genetics Center or Regeneron Pharmaceuticals. L.K. is an employee of BioAge Labs and holds share options. J.S.W. has received research support from Bristol Myers Squibb and has acted as a consultant for MyoKardia, Pfizer, Foresite Labs, Health Lumen, and Tenaya Therapeutics.

## Web resources

OMIM, https://www.omim.org

## Supplemental information

Document S1. Participant information sheet and consent form for return of results

Table S1. All actionable variants reported by our ClinVar pipeline

Document S2. Article plus supplemental information
