# Supplementary material for: Actionable genetic variants in 4,198 Scottish participants from the Orkney and Shetland founder populations and implementation of return of results
Source: Am J Hum Genet. 2025 Mar 14;112(4):793–807. doi: 10.1016/j.ajhg.2025.02.018 (PMC12081267; doi:10.1016/j.ajhg.2025.02.018)
Supplement: Document S1. Participant information sheet and consent form for return of results [file mmc1.pdf]

The American Journal of Human Genetics, Volume *112*

Supplemental information

Actionable genetic variants in 4,198 Scottish participants from the Orkney and Shetland founder populations and implementation of return of results

Shona M. Kerr, Lucija Klaric, Marisa D. Muckian, Kiera Johnston, Camilla Drake, Mihail Halachev, Emma Cowan, Lesley Snadden, John Dean, Sean L. Zheng, Prisca K. Thami, James S. Ware, Gannie Tzoneva, Alan R. Shuldiner, Zosia Miedzybrodzka, and James F. Wilson

Participant Information Sheet - Return of Results

A few years ago, you took part in one of our research studies. This was in either the Orkney Complex Disease Study (ORCADES) or the Viking Health Study – Shetland. We invited you to complete a questionnaire and visit clinics, so we could take samples and collect data for genetic research.

Since then, new permissions and funding have allowed us to provide you with the chance to have some genetic results returned, if they would benefit you. We'll only contact you about these results if we find any that are relevant to your health during our research.

This is voluntary. You can keep taking part if you don't wish to have results returned.

The following information will tell you about **why** we might want to return your results and **what it could mean** for you. If you have

any questions after reading this information sheet you can call us on **0131 651 8557** or email us at viking@ed.ac.uk.

What is genetic research?

Genes are made up of DNA, which is the material found in humans and all other living things. We share more than 99% of our DNA with all other people. However, the small differences help define each person's features and chances of disease.

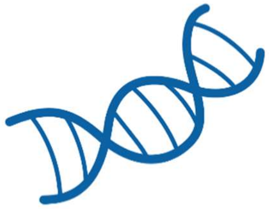

Genetic research is the study of genes and how these small differences in genes can affect people's health and well-being. Sometimes, we may find 'actionable' genetic results that we'll want to tell you about.

What is an 'actionable' genetic result?

'Actionable' genetic results are gene changes you may have that are linked to a condition or disease. We'd like to tell you about some of these changes, that the NHS can treat or prevent.

These gene changes are uncommon. We expect that about one or two in every hundred people will have them. We'll only let you

know about these results if you agree for us to do so. You can change your mind about this at any time.

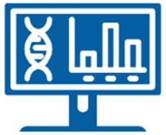

The NHS keeps lists of genes in which there could be changes that we can let volunteers know about. This list will change over time, as science gets better at predicting which gene changes cause disease.

Why do you want to return my results?

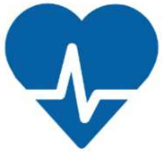

If we find information as part of our research that could improve your health, we feel you should be given the chance to know about it. You may learn about a gene that you didn't realise was impacting your health or could impact your future health. Once you and your doctors are aware of it, steps can be taken to prevent or reduce the impact of this gene on your future health.

When will I receive feedback?

We plan to begin the feedback of results as soon as possible. However, it could be a considerable amount of time before you receive any feedback.

You may not get any feedback because only those with 'actionable' genetic results will hear from us. New research means that results could continue to be made well into the future. We will try to continue to return results for as long as possible.

Do I need to have my results returned to keep taking part?

No, you can stay in the study if you don't want feedback. If you don't agree to feedback on 'actionable' genetic results, your involvement will not be affected, in any way.

What happens if there is an 'actionable' result?

We will discuss any potentially important results with the NHS Clinical Genetics Service in Aberdeen. You'll be asked to provide a new sample of blood to confirm the initial test result was accurate. An NHS genetics expert will then provide you with your results and they'll discuss what it means for you. They'll support, advise and answer any questions you have.

What could this mean for my family?

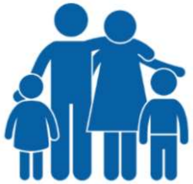

An 'actionable' genetic result in your DNA may suggest that other family members have also inherited the same gene change. NHS experts on

genetic findings in families would confidentially discuss the benefits and risks of the genetic testing with you.

What's the benefit of agreeing to return of results?

If you have an 'actionable' result returned, you'll have the chance to get treatment or prevention for the condition, through the NHS. You will be able to discuss the result with an NHS genetics expert who can answer any questions you may have.

Why don't you give feedback directly to volunteers?

Any findings need to be carefully checked and understood by NHS experts before they can feedback to you. When you receive feedback they will advise you on the next steps.

If I don't get results, am I free of any condition?

No, you should always contact your GP if you have **any** concerns about your health. Our work mainly focuses on genetic research that does not involve looking for 'actionable' genetic results.

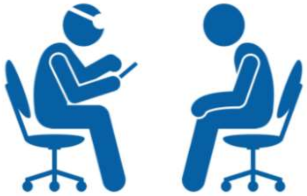

We're only offering to return a limited set of genetic findings.

What are the risks to having my results returned?

You may become anxious if you learn that you have an increased chance of disease, due to your genetics. It's natural to be concerned about a positive result and this is why you would be able to receive advice from trained NHS staff. You can be assured that we won't inform you of any finding that can't have actions taken to resolve or reduce its impact.

Has this study been approved?

Our research has been reviewed by an independent group of people, called a Research Ethics Committee. They're here to protect your safety, rights, wellbeing and dignity. This project amendment was reviewed and given a favourable opinion by the South East Scotland Research Ethics Committee of NHS Lothian.

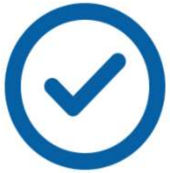

I'm still not sure, where can I find more information?

You can find out further information on return of results on our website:

www.ed.ac.uk/viking/volunteer-for-viking/faqs/return-of-results

You can also read more about how we protect your privacy here:

https://www.ed.ac.uk/viking/privacy-notice

How do I contact you?

You can contact us if you have any questions, concerns or complaints about anything to do with our study:

By Phone: **0131 651 8557** Mon – Fri 9.00 – 17.00 (answerphone outside working hours)

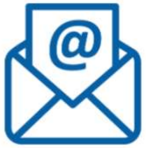

By Email: viking@ed.ac.uk

On Social Media: www.twitter.com/vikinggenes

www.facebook.com/vikinggene

www.instagram.com/viking_genes

Or, you can write to us at:

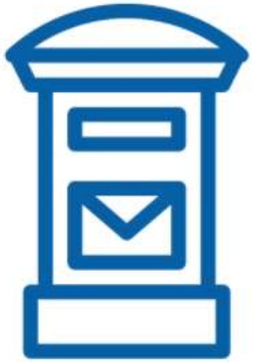

VIKING Genes, MRC Human Genetics Unit
Institute of Genetics and Cancer
The University of Edinburgh
Western General Hospital
Crewe Road South
Edinburgh, EH4 2XU, Scotland

Independent Genetic Advisor

Prof. Zosia Miedzybrodzka has agreed to be the independent genetic advisor of the study. Zosia will be able to answer any specific questions about genetics in VIKING Genes that you may have. You can contact Zosia using her details below:

Email: zosia@abdn.ac.uk

Prof. Zosia Miedzybrodzka,
Medical Genetics Group,
University of Aberdeen,
Polwarth Building,
Aberdeen, AB25 2ZD, Scotland

Independent advisor/complaints contacts

If you would like to speak to someone about the study who is not part of the research team, please contact Prof. Sarah Wild on 0131 651 1630 or email sarah.wild@ed.ac.uk

If, after discussing any issues with the research team, you wish to make a formal complaint about the study, please contact the University of Edinburgh's Research Governance team via email at: researchgovernance@ed.ac.uk

Participant ID:

CONSENT FORM
"VIKING"

Please **initial** box

1. I confirm that I have read and understand the information sheet (2022-08-17 Participant Information Sheet RoR Version Number 1.0) for the above study. I have had the opportunity to consider the information, ask questions and have had these questions answered satisfactorily. ☐

2. I agree to have actionable results returned to me, via NHS geneticists, as described in the Participant Information Sheet. You do not have to agree to this to remain part of the study. ☐ YES ☐ NO

3. I understand that my participation is voluntary and that I am free to withdraw at any time, without giving any reason and without my medical care and/or legal rights being affected. ☐

4. I agree to take part in the return of results component of the Viking Genes study. ☐

Name of Person Giving Consent	Date	Signature

VIKING Genes

Participant Information Sheet

Return of Results – Next of Kin

New permissions have allowed us to provide the next of kin of participants in our Viking Genes studies with the chance to have some genetic results returned, if they would be of benefit.

We'll only inform you about these results if we find any that might have been relevant to the health of your next of kin and therefore potentially their family, during our research.

The following information will tell you about why we might want to return results, and what it could mean for their family.

If you have any questions after reading this information sheet, you can call us on **0131 651 8557** or email us at viking@ed.ac.uk

What is genetic research?

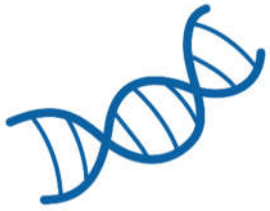

Genes are made up of DNA, which is the material found in humans and all other living things. We share more than 99% of our DNA with all other people. However, the small differences help define each person's features and chances of disease.

Genetic research is the study of genes and how these small differences in genes can affect people's health and well-being.

What is an 'actionable' genetic result?

Sometimes, we may find 'actionable' genetic results that we'll want to share. 'Actionable' genetic results are gene changes a person may have that are linked to a treatable condition or disease, for example an inherited predisposition to heart disease or cancer. We'd like to tell you as next of kin about some of these changes, if we find them.

These gene changes are uncommon. We expect that about one or two in every hundred people will have them. We'll only let you know

about these results about your next of kin if you agree for us to do so. You can change your mind about this at any time.

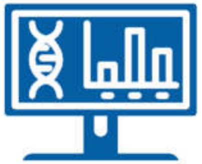

Medical professionals keep lists of genes in which there could be changes that we can let volunteers, or their next of kin, know about. This list will change over time, as science gets better at predicting which gene changes cause disease. We're only offering to return a limited set of genetic findings, but we will try to continue to return results for as long as possible.

Why do you want to return these results?

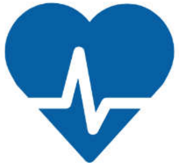

If we find information as part of our research that could improve the health of the family of one of our volunteers, we feel you as next of kin should be given the chance to know about it. Once you and your doctors are aware of it, steps can be taken to prevent or reduce the impact of the gene on the future health of the family.

When will I receive feedback?

We have begun the feedback of results, and if an actionable finding is made, we would aim to contact you by letter as soon as possible, if you consent as next of kin. You will most likely not get any

feedback, because only those with 'actionable' genetic results will hear from us.

What happens if there is an 'actionable' result?

We will discuss any potentially important results with the NHS Clinical Genetics Service in Aberdeen. An NHS genetics expert will assess what it means for the family of your next of kin, before we would send you a letter.

What could this mean for the family?

If you have an 'actionable' result returned, the letter will include

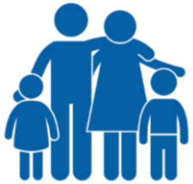

contact details for the NHS clinical genetics team. If you get in touch with them, the risk to the family of your next of kin will be explained by an NHS genetics expert. They'll support, advise and answer any questions you may have. If your next of kin carried an actionable finding, it does not mean their children will definitely carry this genetic variant.

NHS experts on genetic findings in families would confidentially discuss the benefits and risks of genetic testing in the NHS. They will also advise you on the next steps family members can take,

MRC Human Genetics Unit · THE UNIVERSITY of EDINBURGH · usher institute · NHS Lothian · UNIVERSITY OF ABERDEEN

including treatment and prevention of the condition, where necessary.

What are the risks to having results returned?

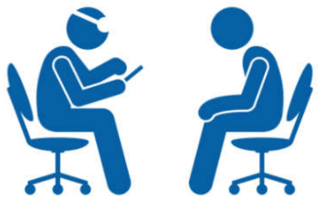

You may become anxious if you learn that your next of kin's family may have an increased chance of disease, due to their genetics. It's natural to be concerned about a positive result and this is why you would be able to receive advice from trained NHS staff. You can be assured that we won't inform you of any finding that can't have actions taken to resolve or reduce its impact.

Has this study been approved?

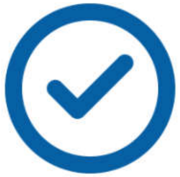

Our research has been reviewed by an independent group of people, called a Research Ethics Committee. They're here to protect your safety, rights, wellbeing and dignity. This project amendment was reviewed and given a favourable opinion by the South East Scotland Research Ethics Committee of NHS Lothian.

Where can I find more information?

You can find out further information about return of results on our website:

www.ed.ac.uk/viking/volunteer-for-viking/faqs/return-of-results

You can also read more about how we protect the privacy of our volunteers here:

https://www.ed.ac.uk/viking/privacy-notice

How do I contact you?

You can contact us if you have any questions, concerns or complaints about anything to do with our study:

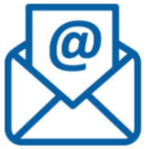

By Phone: **0131 651 8557** Mon – Fri 9.00 – 17.00

By Email: viking@ed.ac.uk

Or, you can write to us at:

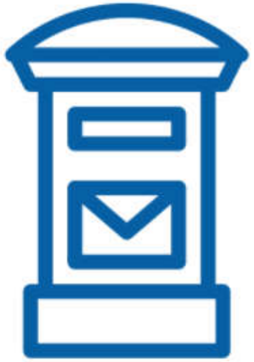

VIKING Genes, MRC Human Genetics Unit
Institute of Genetics and Cancer
The University of Edinburgh
Western General Hospital
Crewe Road South
Edinburgh, EH4 2XU, Scotland

Independent Genetic Advisor

Prof. Zosia Miedzybrodzka has agreed to be the independent genetic advisor of the study. Zosia will be able to answer any specific questions about genetics in VIKING Genes that you may have. You can contact Zosia using her details below:

Email: zosia@abdn.ac.uk

Prof. Zosia Miedzybrodzka
Medical Genetics Group,
University of Aberdeen,
Polwarth Building,
Aberdeen, AB25 2ZD, Scotland

Independent advisor/complaints contacts

If you would like to speak to someone about the study who is not part of the research team, please contact Prof. Sarah Wild on 0131 651 1630 or email sarah.wild@ed.ac.uk

If, after discussing any issues with the research team, you wish to make a formal complaint about the study, please contact the University of Edinburgh's Research Governance team via email at: researchgovernance@ed.ac.uk

MRC Human Genetics Unit THE UNIVERSITY of EDINBURGH usher institute NHS Lothian UNIVERSITY OF ABERDEEN
